# Categorical frequency judgments as effective ensemble judgments for object features

**DOI:** 10.1038/s41598-025-93760-5

**Published:** 2025-03-27

**Authors:** Oakyoon Cha

**Affiliations:** 1 Department of Psychology, Sogang University, Seoul, 04107 Republic of Korea; 2 Department of Psychology, Sungshin Women’s University, Seoul, 02844 Republic of Korea

## Abstract

The present study explored the potential of categorical frequency judgments as effective ensemble judgments, motivated by the observation that most studies on ensemble judgments have focused on univariate statistics, such as mean and variance. However, these univariate statistics may not fully capture the complexity of real-world tasks that require judgments on complex object features. In such cases, categorical statistics like mode (the most frequent instance in a set) and diversity (the number of different instances in a set) may provide more relevant information. For instance, when a speaker enters an auditorium and scans her audience, relative frequencies of different emotional expressions could be more useful than the representation of the average face with a potentially faint expression. Study 1 examined the relationship between mode judgment and diversity comparison in facial identities, while Study 2 extended the examination of mode judgments across different object categories (faces and blobs). The results indicate that categorical frequency judgments share behavioral variability across tasks and object categories, supporting their potential as effective ensemble judgments. Future research may explore how these categorical frequency judgments interact with univariate statistical judgments to enhance our understanding of ensemble judgments.

## Introduction

Imagine a golfer leaning forward to read the greens for a putt. Rather than analyzing the orientation of each individual blade of grass, she intuitively judges the average orientation of blades in clusters and the overall pattern these averages create. People often perform visual tasks that could be better described as judgments on ensemble properties (e.g., average, variability) rather than on visual features of individual items, such as selecting the freshest fruit basket and estimating the change of seasons by looking at the average color of leaves (for review, see^1^). Interest in ensemble judgments gained traction after Chong and Treisman^2^ demonstrated that people can judge the mean size of circles presented for only 50 ms. These judgments seem to circumvent limitations of attention^3,4^ and working memory^5^, providing a means for processing rich visual scenes populated with numbers of objects^6^. In support of this idea, studies have shown that people can judge various ensemble properties helpful for summarizing rich visual information, including mean size^2,7^, size variance^8,9^, mean orientation^10,11^, distribution of orientations^12^, mean color^13,14^, distribution of colors^15^, etc.

Ensemble judgments extend to more complex visual features (e.g., facial expression and gender^16^) and even abstract concepts (e.g., life-likeness^17^), suggesting a broader applicability in visual tasks. For example, a speaker entering an auditorium might quickly judge whether the audience appears interested or bored. The ability to assess ensemble properties from faces offers clear evolutionary and social advantages. People are adept at judging various ensemble properties from a group of faces, such as mean emotion and gender^16^, mean attractiveness^18^, and mean gaze direction^19^. Interestingly, people can also judge ensemble properties that do not have clear evolutionary or social advantages, such as the diversity of race and gender^20^, facial identity and car models^21^, as well as the mean shape of bird species, airplanes, and car models^22^. The broad range of object features that can be judged with ensemble properties suggests that this ability can be used to summarize visual information across various categories of objects.

It is natural to question whether ensemble judgments for simple features and complex object features rely on shared mechanisms. Haberman and colleagues^23^ investigated correlations among ensemble judgments of simple features (orientation and color) and complex object features (identity and expression of faces). They found that participants’ ensemble judgment performance was correlated within the same level (i.e., within simple features and within complex object features) but not across different levels, suggesting at least two independent levels of processing, one for simple features and the other for complex object features. In contrast, Chang and colleagues^24^ measured participants’ ensemble judgment performance with a range of tasks (mean estimation, diversity comparison, mean matching) and visual features (simple features: orientation, brightness, aspect ratio; complex object features: bird species, Transformer robot identity [robot character from popular media], Ziggerin shape [novel object]). Using a latent variable approach, they found that participants’ ensemble judgment abilities for simple and complex object features were highly correlated (*r* = .90), suggesting shared mechanisms for ensemble judgments across these features. Chang and colleagues attributed the different results to the use of multivariate analysis with a broad range of tasks, which allows better estimates for latent variables^25^.

Studies on ensemble judgments, including both Haberman et al.^23^ and Chang et al.^24^, typically conceptualize ensemble judgments as estimation of univariate statistics, such as mean and variance. This conception works well for simple visual features, such as estimating the average color of leaves. However, this approach may not always work as well with complex object features. For instance, when a speaker gauges the mood of her audience, it would be challenging to represent an average face and discern whether the facial expression of that face leans slightly more toward interest or boredom. A single average expression, calculated across multiple dimensions such as the eyes, mouth, and eyebrows, may dilute distinct emotional cues, making it less informative. In contrast, categorical frequency judgments, such as perceiving the prevalence of interested and bored faces, allow the speaker to assess the relative contributions of specific emotional expressions in the crowd. Univariate statistics like the mean require combining values across dimensions into a single estimate, which can mask critical differences between objects. To avoid this, many studies used a limited set of faces generated from linear morphs between pairs of faces (e.g.,^23,26^), which only covers a limited portion of the expansive object space. This limited coverage could reduce the generalizability of findings to real-world scenarios involving more diverse and complex object spaces. Only a handful of studies on ensemble judgments^17,21,24,27–29^ have used non-morphed objects sampled from a broader object space. To better understand the relationship between ensemble judgments for simple features and complex object features, it is important to use ensemble judgment tasks that are more representative of everyday scenarios, especially for complex object features.

The goal of the present study is to investigate whether categorical frequency judgments for object features can be considered as effective ensemble judgments. Note that while categorical frequency is not inherently multivariate, complex objects often occupy multidimensional object spaces, making their features multivariate. In this study, I conceptualized the mode (the most frequent instance in a set) as a counterpart to the mean in the categorical frequency context, as both represent central tendency within a group. Similarly, I treated diversity (the number of different instances in a set) as analogous to variance, as both reflect variability within a group. These are conceptual analogies rather than strict mathematical equivalences. Firstly, I examined whether performance on the mode judgment and diversity judgment tasks correlate. Such a correlation would suggest shared mechanisms for different types of ensemble judgments in the categorical frequency context. Secondly, I investigated the shared variability in mode judgments across different object features (identity of face, shape of random blob). Random blobs were selected as simple stimuli with features spanning multiple dimensions. A correlation between these judgments would suggest shared mechanisms underlying categorical frequency judgments across different object categories.

## Results

**Fig. 1:**
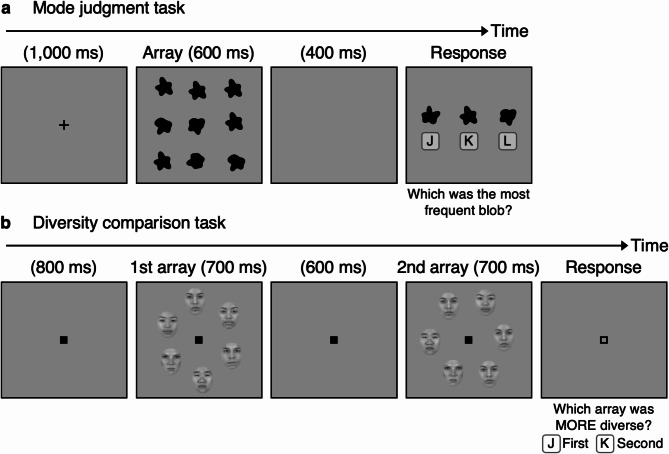
Procedures for the mode judgment task (**a**) and diversity comparison task (**b**). For presentation purposes, fixation points and key labels (J, K, L) are not drawn to scale. The face images in (**b**) are sourced from the Chicago Face Database^48^, with appropriate permissions obtained from the copyright holders. In this example, the correct answer is “K” for the mode judgment task and “J” for the diversity comparison task. In the second array of (**b**), the face images at 11, 3, and 5 o’clock are identical, making the second array less diverse.

Study 1 examined the correlation between mode judgment task performance (MJ-Face) and diversity comparison task performance (DC-Face) for faces, while Study 2 examined the correlation between mode judgment performance for faces (MJ-Face) and random blobs (MJ-Blob). In the mode judgment task, participants selected the most frequent identity/shape from three alternatives based on the preceding array of nine objects (Figure 1a). In the diversity comparison task, participants determined which of two sequentially presented arrays of six objects had a greater number of different identities/shapes (Figure 1b). Informed consent was obtained from all participants. In both studies, participants also completed the Cambridge Face Memory Task (CFMT)^30^. Variability due to participants’ face recognition ability (CFMT performance) was statistically removed from mode judgment and diversity comparison scores to control for abilities not directly related to ensemble judgments. Since the mode judgment task posed similar task requirements (comparison between mental representation and given alternatives) within the same task format (3-alternative forced choice) as in CFMT, controlling for CFMT performance was expected to remove behavioral variability associated with the task and, additionally, more general factors such as motivation and working memory.

### Correlation between mode judgment and diversity comparison for faces

Data from 79 participants were analyzed in Study 1. The means, standard deviations, and Spearman-Brown corrected split-half reliabilities of accuracy scores for the three tasks are shown in Table 1. The table also includes split-half reliabilities for MJ-Face and DC-Face performance measures after controlling for CFMT performance (MJ-Face and DC-Face residuals). All split-half reliabilities, including those for the residuals, were acceptable (> .7).

**Table 1:** Descriptive statistics for the three tasks used in Study 1. MJ-Face and DC-Face residuals were calculated by regressing out CFMT performance from MJ-Face and DC-Face performance, respectively.

Task/measure	Mean accuracy (SD)	Split-half reliability
MJ-Face	.655 (.096)	.772
DC-Face	.673 (.086)	.715
CFMT	.704 (.170)	.894
MJ-face residual	–	.713
DC-face residual	–	.703

**Fig. 2:**
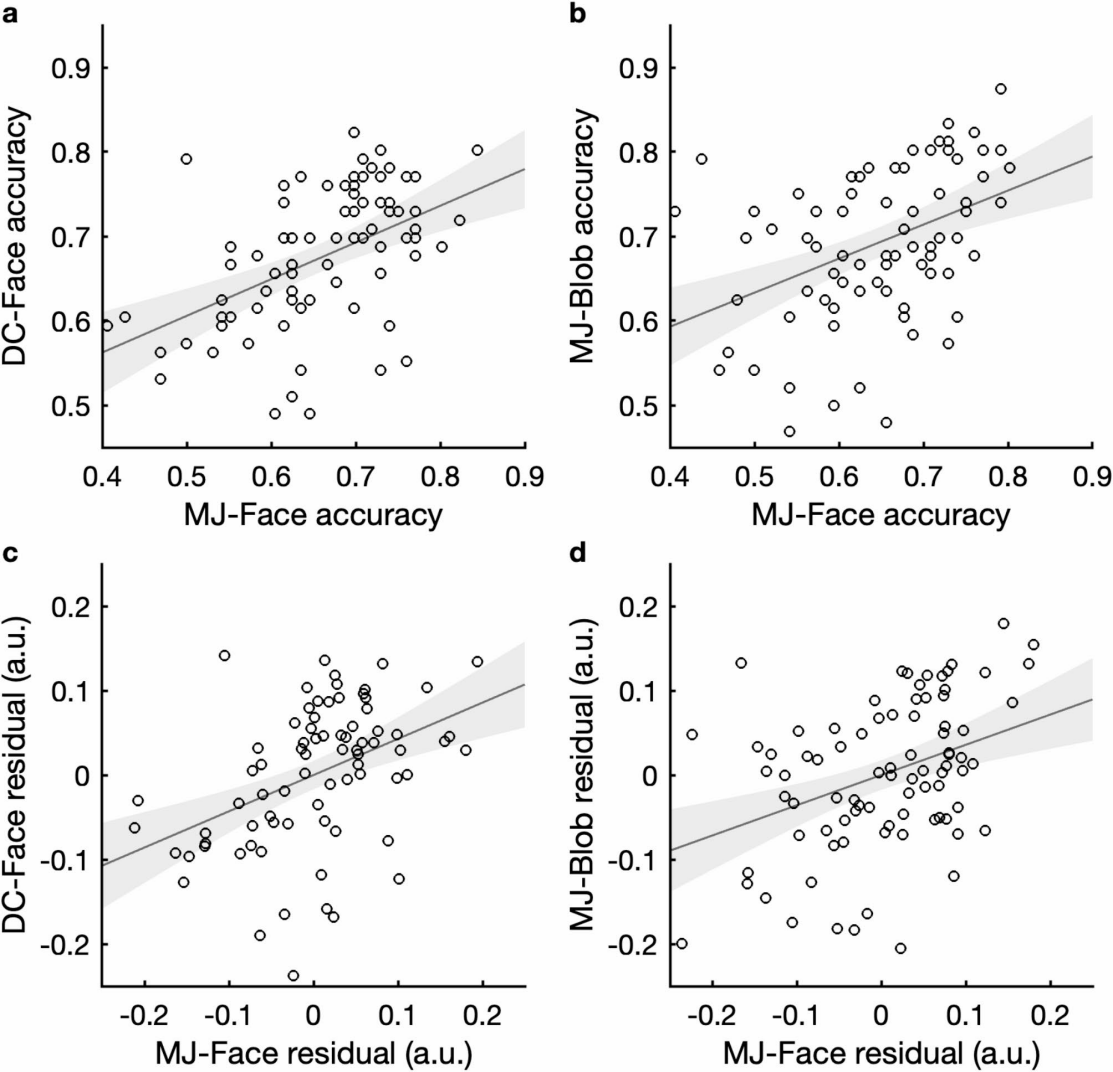
Correlations and partial correlations between MJ-Face and DC-Face performance in Study 1 and between MJ-Face and MJ-Blob performance in Study 2. Panels (**a**,**b**) display correlations between accuracy scores, while panels (**c**,**d**) display partial correlations controlling for CFMT performance. Each datapoint represents an individual participant’s accuracy score in panels (**a**,**b**) or residual accuracy score in panels (**c**,**d**). The light gray areas around the regression lines indicate 95% confidence intervals, with the regression lines shown in dark gray.

Bayesian analyses provided very strong evidence^31,32^ for a positive correlation between CFMT and MJ-Face performance (*r* = .395, BF_10_ = 83.216), while favoring the absence of a correlation between CFMT and DC-Face performance (*r* = .206, BF_10_ = 0.717). Decisive evidence was found for a positive correlation between DC-Face and MJ-Face performance (*r* = .488, BF_10_ = 3783.414, Figure 2a). Importantly, this correlation remained decisive even after controlling for CFMT performance (*r*_*p*_ = .452, BF_10_ = 749.681, Figure 2c), suggesting shared mechanisms underlying the two different types of ensemble judgments in the categorical frequency context.

### Correlation between mode judgment for faces and random blobs

Data from 85 participants were analyzed in Study 2. The means, standard deviations, and Spearman-Brown corrected split-half reliabilities of accuracy scores for the three tasks are shown in Table 2. The table also includes split-half reliabilities for MJ-Face and MJ-Blob performance measures after controlling for CFMT performance (MJ-Face and MJ-Blob residuals). All split-half reliabilities, including those for the residuals, were acceptable (> .7).

**Table 2:** Descriptive statistics for the three tasks used in Study 2. MJ-Face and MJ-Blob residuals were calculated by regressing out CFMT performance from MJ-Face and MJ-Blob performance, respectively.

Task/measure	Mean accuracy (SD)	Split-half reliability
MJ-Face	.640 (.102)	.797
MJ-Blob	.690 (.092)	.774
CFMT	.703 (.172)	.901
MJ-Face residual	–	.769
MJ-Blob residual	–	.758

Bayesian correlation analyses supported positive correlations among all three measures: moderate evidence for a positive correlation between CFMT and MJ-Face performance (*r* = .303, BF_10_ = 6.797) and between CFMT and MJ-Blob performance (*r* = .284, BF_10_ = 4.152), and decisive evidence for a positive correlation between MJ-Face and MJ-Blob performance (*r* = .446, BF_10_ = 1093.255, Figure 2b). The positive correlations between CFMT performance and the other two measures could be attributed to similarities in task requirements, task format and/or stimuli, justifying the use of CFMT to control for variability unrelated to ensemble judgments. Bayesian evidence for the correlation between MJ-Face and MJ-Blob performance remained decisive after controlling for CFMT performance (*r*_*p*_ = .394, BF_10_ = 152.838, Figure 2d), suggesting shared mechanisms for mode judgments across different categories of objects.

An additional exploratory analysis was conducted to test whether CFMT performance was more strongly correlated with MJ-Face performance than with MJ-Blob performance. A stronger correlation between CFMT and MJ-Face performance would support the idea that the shared variability between MJ-Face and CFMT arose from task requirements, task format, and the cateogry of stimuli, whereas the shared variability between MJ-Blob and CFMT would be unrelated to the cateogry of stimuli. The BFpack R package^33^ was used to compare the hypothesis that the correlation was greater between MJ-Face and CFMT performance than between MJ-Blob and CFMT performance (H1) and the hypothesis that the two correlations were comparable (H2). This analysis yielded moderate evidence in favor of H1 (BF_12_ = 6.356).

## Discussion

The present study investigated whether categorical statistics, specifically mode (the most frequent instance in a set) and diversity (the number of different instances in a set), could be considered as effective properties for ensemble judgments. The premise was that complex object features, such as facial identity/expression, bird species, or car model, could be better judged as an ensemble based on the frequency of object features rather than on a visual average. For example, reading the atmosphere from a crowd might be better achieved by estimating relative frequencies of different facial expressions rather than by discriminating the potentially faint expression on an average face. Study 1 found that different types of frequency judgments (mode and diversity of faces) shared behavioral variability after controlling for face recognition ability. Additionally, only mode judgment, not diversity comparison, shared variability with face recognition, suggesting shared components in the judgments for the most frequent face (MJ-Face task) and those for a memorized face (CFMT). Study 2 extended the findings of Study 1 by revealing shared variability between mode judgments across different object categories (faces and blobs). These results suggest shared mechanisms responsible for judgments on categorical statistics.

Both the MJ-Face and DC-Face tasks in the present study included arrays with repeated face images. While repetitions of the same face in a crowd are uncommon, they do not undermine the tasks’ relevance for investigating categorical frequency judgments. First, highly similar stimuli are often encountered in other contexts, such as herds of cattle or clusters of leaves viewed from a distance, where humans likely employ categorical frequency judgments. Second, repeated faces are often found in cases like performance or campaign posters, where ensemble judgments can effectively utilize the redundancy of repeated images. These examples underscore the ecological validity of the MJ-Face and DC-Face tasks for studying categorical frequency judgments.

In the present study, the stimulus array remained on the screen for 600 ms (mode judgment task) or 700 ms (diversity comparison task), durations that allow participants to make multiple eye movements during array presentation. Eye movements could be prevented with very short stimulus durations, as used in some experimental studies (for review, see^1^). However, in an individual differences study, longer presentation times are not necessarily problematic. As long as each participant consistently applies their own viewing strategy (e.g., making eye movements in a particular pattern), this should not affect the reliability of individual differences measurements. If participants employed different eye movement strategies across tasks, this would likely reduce observed correlations by decreasing the overlap in mechanisms used across tasks, rather than inflating them. Thus, while eye movements could influence task performance, they are unlikely to undermine the study’s conclusions about shared variability in categorical frequency judgments.

One interesting finding in the present study is the evidence against a correlation between DC-Face and CFMT performance (BF_10_ < 1), despite both tasks being associated with facial identity. This suggests that the DC-Face task does not engage facial identity comparison, unlike the MJ-Face task, which showed a correlation with CFMT performance, likely due to its reliance on such comparisons. For univariate statistical judgment of simple features, studies have shown that participants can form a representation of feature distribution from a group of objects^12,15,34^. Perhaps in the diversity comparison task, participants relied on a representation of object feature distribution similar to that of simple features, but spanning multiple dimensions within a complex object space.

Although the present study found correlations across different types of frequency judgments and object categories, concluding that categorical frequency judgments rely on a “common statistical processor” would be premature. Research on univariate ensemble judgments has yielded mixed findings regarding shared mechanisms for mean and variance estimations. For example, adaptation to orientation variance has been shown to reduce sensitivity to mean orientation and adaptation to specific mean orientation biased orientation variance estimation, suggesting shared mechanisms^35^. Conversely, Hansmann-Roth et al.^36^ found evidence of shared variability (or “common noise source”) only when participants engaged in explicit ensemble judgments. Another study using dual tasks for mean and variance estimation found no common variability in performance^37^, but correlations emerged when the tasks were performed in separate blocks with matched task requirements^8^. Findings on ensemble judgments across different features have also been inconsistent. While some studies suggest distinct mechanisms for ensemble judgments of low- and high-level features (e.g.,^23)^, others suggest shared mechanisms (e.g.,^24,27^).

Given these mixed findings, exploring computational or physiological models for univariate ensemble judgments may provide insights into the mechanisms underlying categorical frequency judgments. One class of models assumes activations in a feature space. Utochkin et al.^38^ proposed a two-layer model with one layer for individual feature representations and another for pooling these activations. Robinson and Brady’s model^39^ uses summed activations from individual items to form ensemble representations. In these models, pooled or summed activations create a distribution over a feature space, which can be used to estimate mean and variance. Extending these ideas to categorical frequency judgments, one could assume that individual objects elicit specific activation patterns in relevant feature spaces. The visual system might sum these activations to identify the object representation aligning most closely with the dominant pattern for mode judgments, or consider the number of roughly matching representations for diversity judgments. Another potential mechanism involves models for texture perception. Cain and Cain^40^, for instance, suggested that ensemble judgments for mean size and mean emotion could be derived from image statistics. Because image statistics are mostly build from low-level features, these models might apply to both univariate ensemble judgments and categorical frequency judgments. Finally, mechanisms based on numerosity perception^41,42^ offer a third possibility. If repeated objects can be grouped perceptually, numerosity perception for each group might facilitate categorical frequency judgments. However, recent findings suggesting that grouping by feature conjunction is ineffective during ensemble judgments^43^ cast doubt on this possibility.

Building on these models, future research should explore how categorical frequency judgments are made for univariate features and how these judgments interact with univariate statistical judgments. Categorical and univariate statistics, such as mode and mean, can differ for univariate features, and different tasks may require judgments on one type of statistic over the other. For example, you might need to know the mean number of people per table to determine the amount of food required, but the mode number of people per table to decide how many sets of silverware to prepare for a table. Although no studies have specifically investigated frequency judgments for univariate features to my knowledge, insights can be drawn from previous research on outlier discounting. Studies on ensemble judgments have found that outlier features are often discounted when participants make judgments about univariate statistics, such as the mean^44,45^. In these studies, outliers were determined based on their deviation from the mean, but these features also had to be rare to qualify as outliers. Categorical frequency judgments could provide useful information for detecting these outliers. Investigating the relationship between categorical and univariate statistical judgments could enhance our understanding of ensemble judgments. Finally, research on diversity judgments across various object categories could provide insights into whether categorical frequency judgments rely on shared mechanisms.

In conclusion, the present study found shared behavioral variability in categorical frequency judgments for different statistics (mode and diversity, Study 1) and across different object categories (faces and blobs, Study 2). These results suggest that categorical frequency judgments can be considered effective ensemble judgments. However, understanding how these judgments interact with univariate statistical judgments requires further investigation.

## Methods

### Participants

All participants were recruited via Prolific (https://www.prolific.com) with two constraints: they had to be over 18 years old and use US IP addresses. These constraints ensured that participants understood the directions given in English during the study. The sample sizes for both studies were determined using the Sequential Bayes Factor (SBF) design^46^. In the SBF design, data collection and Bayesian analysis are conducted simultaneously after the sample size reaches a predetermined minimum until predetermined criteria are met. For both studies, the minimum sample size was 75, and the criterion was a Bayes factor of interest either larger than 3 or smaller than 1/3 (Study 1 preregistered at https://osf.io/gvjnk, Study 2 preregistered at https://osf.io/yj6pt). Data were collected from 91 participants in Study 1 and 90 participants in Study 2. Data from participants who performed no better than chance (accuracy below the one-sided 90% confidence interval of a binomial distribution) and had a trial-by-trial correlation with trial difficulty (proportion of correct responses across participants who performed above chance) lower than .15 in any of the three tasks were excluded. The final sample sizes after the exclusion were 79 for Study 1 (age: M=35.4, SD=13.5; 38 men, 36 women, 2 gender variants, and 3 who preferred not to answer) and 85 for Study 2 (age: M=34.6, SD=11.2; 42 men, 41 women, 1 gender variant, and 1 who preferred not to answer).

### Apparatus

Tasks were created using HTML and JavaScript with the jsPsych library version 7.2.1^47^ and were hosted on the Cognition platform (https://www.cognition.run). Participants used a web browser on a desktop or laptop computer of their choice. Since both viewing distance and stimulus sizes in visual angle varied across participants, all stimulus sizes and configurations are reported in pixels.

### Stimuli

Face images used in the MJ-Face and DC-Face tasks were generated as follows. Face images with neutral expressions were selected from the Chicago Face Database (CFD)^48^. Custom MATLAB scripts detected facial landmark points from the selected images using OpenFace version 2.2.0 (https://github.com/TadasBaltrusaitis/OpenFace), and then generated facial contours from these landmark points. The image area outside the facial contour was trimmed to remove non-facial features (e.g., hair, beard). MATLAB scripts for this procedure are available from the GitHub repository at https://github.com/dcantlab/face-datasets (see CFD-cutout images). Among the trimmed images, six were selected from each CFD race and gender category (Asian Female, Asian Male, Black Female, Black Male, Latino Female, Latino Male, White Female, White Male) based on their facial contour similarity to the average facial contour of all faces. Half of these images (3 images × 8 categories) were used in the MJ-Face task, and the other half in the DC-Face task.

Blob images used in the MJ-Blob task were generated using custom MATLAB scripts. The process began by creating 240 one-dimensional white-noise arrays, which were then filtered with a low-pass filter (cutoff frequency: 6 Hz) and a notch filter (attenuation frequency: 0 Hz). The values in each filtered array were used to modulate the radius around the circumference of a unit circle, resulting in one blob image per array. Blob images were then clustered into six sets using k-means clustering based on the spectral power distributions of the filtered arrays. Four images closest to each cluster center were selected to comprise the stimulus sets. MATLAB scripts for this procedure are available from the GitHub repository at https://github.com/dcantlab/randstim-datasets (see splash-categorical images). Each array used in the MJ-Blob task contained no more than one blob from the same set, ensuring that all blobs in the array display could be easily distinguished.

In the mode judgment tasks (MJ-Face and MJ-Blob, Figure 1a), all stimuli, including the three alternatives in the response display, were presented within a gray rectangle (640×560 px) that remained visible throughout the task. The array display consisted of nine object images, each centered within a cell (168×168 px) of a virtual 3×3 grid, with horizontal and vertical jitters of ±12 px. The number of objects, their configuration, and their presentation duration were determined in a separate pilot experiment to ensure sufficient individual differences in the accuracy measures. The response display presented three object images, each labeled with the corresponding key (J, K, L), with the response directions (“Which was the most frequent face/blob?”) shown below the gray rectangle.

In the diversity comparison task (DC-Face, Figure 1b), all stimuli were presented within a gray rectangle (640×560 px), similar to the mode judgment task. The array display consisted of six face images, each positioned at equal intervals along the circumference of a virtual, vertically elongated ellipse (332×365 px). The number of faces, their configuration, and their presentation duration matched those used in a previous study^21^ to ensure sufficient individual differences in accuracy measures. It is important to note that participants viewed two sets of images (12 faces in total) per trial in the DC-Face task. The center of each face image was placed directly on the ellipse’s boundary, with images spaced 60° apart. Additionally, the position of each face image was jittered horizontally and vertically by ±14 px. The response display showed the empty gray rectangle with the response directions (“Which array was MORE diverse? [J] First array [K] Second array”) below. To ensure participants did not confuse diversity in this task with racial diversity, they were provided with example displays and explicitly informed that, in this study, a greater number of different facial identities indicated a more diverse group.

### Procedures

In Study 1, participants completed the DC-Face task, MJ-Face task, and CFMT, in that order. In Study 2, participants performed the MJ-Blob task, MJ-Face task, and CFMT, also in that order. Keeping the task order consistent for all participants helped reduce unwanted variability, which is especially important in individual differences studies^49,50^. In both Studies, all tasks were run in blocks. Before each mode judgment and diversity comparison task, participants received step-by-step instructions along with five practice trials. Before CFMT, participants completed the three practice trials included in the original CFMT^30^. Prior to participation, participants were presented with an informed consent form on a webpage and indicated their consent by clicking the “Accept” button. After providing consent, participants reported their age and selected their gender from the following options: male, female, transgender male, transgender female, other not listed (gender variant), or prefer not to answer. All procedures were approved by the Institutional Review Board of Sungshin Women’s University (SSWUIRB-2021-039) and carried out in accordance with the Declaration of Helsinki.

A trial in the mode judgment tasks (MJ-Face and MJ-Blob, Figure 1a) began with a fixation point in the center of the gray rectangle. After 1000 ms, nine object images were presented on a virtual 3-by-3 grid for 600 ms (array display). Among the nine object images, one image was repeated five times (the most frequent image), another image was repeated twice, and the remaining images depicted all different objects. Although the number of the most frequent image in the array was fixed across all trials, participants were not informed of this. To avoid clustering, no more than three adjacent pairs of the most frequent images appeared in the grid. Following a brief 400 ms interval of an empty gray rectangle, three object images appeared, each labeled with a corresponding response key (response display). Among these images, one depicted the most frequent object (correct response), another depicted an object that appeared once or twice in the array display (50% probability each), and the third depicted an object that was absent from the array display. The images remained until a response was made. After the response, non-guided feedback was provided (“Correct” or “Wrong,” without indicating the correct answer). Participants completed 96 trials in a fixed random order, which helped reduce unwanted variability in individual differences studies^49,50^.

A trial in the diversity comparison task (DC-Face, Figure 1b) began with a fixation point, which remained throughout the trial. After 800 ms, a set of six face images was presented along the circumference of a virtual ellipse for 700 ms (first array display). Following a 600 ms interval with an empty gray rectangle and fixation point, a second set of six face images was presented for 700 ms (second array display). In one array, all six face images were different (more diverse array), while in the other, one face was repeated three or four times (less diverse array). In the less diverse array, no more than two adjacent pairs of repeated images appeared when an image was repeated three times, and no more than three adjacent pairs when an image was repeated four times. After the second array display disappeared, participants indicated which array was more diverse with a key press. Feedback was provided (“Correct” or “Wrong”) after the response. Participants completed 96 trials in a random, fixed order.

### Analysis

Custom MATLAB scripts were used to estimate split-half reliabilities and to regress out CFMT performance from other measures. To estimate split-half reliabilities, 100 pairs of random splits were generated, and the average correlation between pairs in each split was calculated. Bayesian correlation analyses were performed using JASP software^51^, with the default prior (stretched Beta prior width = 1). A custom R script and the BFpack package^33^ were used to compare the strength of correlations among measures.

## Data Availability

The data collected for this study are publicly available on the Open Science Framework repository at https://osf.io/8uraf. The stimuli and program codes used in this study are available from the author (O.C.) upon request.
